# A software system for modeling evolution in a population of organisms with vision, interacting with each other in 3D simulator

**DOI:** 10.18699/VJGB-22-94

**Published:** 2022-12

**Authors:** А.P. Devyaterikov, А.Yu. Palyanov

**Affiliations:** A.P. Ershov Institute of Informatics Systems of the Siberian Branch of the Russian Academy of Sciences, Novosibirsk, Russia Novosibirsk State University, Novosibirsk, Russia; A.P. Ershov Institute of Informatics Systems of the Siberian Branch of the Russian Academy of Sciences, Novosibirsk, Russia

**Keywords:** nervous system, vision system, virtual organism, population, computational modeling, neuroevolution simulator, нервная система, зрительная система, виртуальный организм, популяция, компьютерное моделирование, нейроэволюционный симулятор

## Abstract

Development of computer models imitating the work of the nervous systems of living organisms, taking into account their morphology and electrophysiology, is one of the important and promising branches of computational neurobiology. It is often sought to model not only the nervous system, but also the body, muscles, sensory systems, and a virtual three-dimensional physical environment in which the behavior of an organism can be observed and which provides its sensory systems with adequate data streams that change in response to the movement of the organism. For a system of hundreds or thousands of neurons, one can still hope to determine the necessary parameters and get the functioning of the nervous system more or less similar to that of a living organism – as, for example, in a recent work on the modeling of the Xenopus tadpole. However, of greatest interest, both practical and fundamental, are organisms that have vision, a more complex nervous system, and, accordingly, significantly more advanced cognitive abilities. Determining the structure and parameters of the nervous systems of such organisms is an extremely difficult task. Moreover, at the cellular level they change over time, these including changes under the influence of the streams of sensory signals they perceive and the life experience gained, including the consequences of their own actions under certain circumstances. Knowing the structure of the nervous system and the number of nerve cells forming it, at least approximately, one can try to optimize the initial parameters of the model through artificial evolution, during which virtual organisms will interact and survive, each under the control of its own version of the nervous system. In addition, in principle, the rules by which the brain changes during the life of the organism can also evolve. This work is devoted to the development of a neuroevolutionary simulator capable of performing simultaneous functioning of virtual organisms that have a visual system and are able to interact with each other. The amount of computational resources required for the operation of models of the physical body of an organism, the nervous system and the virtual environment was estimated, and the performance of the simulator on a modern desktop computing system was determined depending on the number of simultaneously simulated organisms.

## Abstract (ru)

Создание компьютерных моделей, имитирующих работу нервных систем живых организмов с учетом их морфологии и электрофизиологии, – один из важных и перспективных разделов вычислительной нейробиологии. При наличии возможности стремятся моделировать не только нервную систему, но и тело, мышцы, сенсорные системы и виртуальную трехмерную физическую среду, в которой можно наблюдать поведение организма и которая обеспечивает его сенсорные системы адекватными потоками данных, изменяющимися в ответ на движение организма. Для системы из сотен или тысяч нейронов еще можно надеяться задать необходимые параметры и получить функционирование нервной системы, более-менее сходное с таковым для живого организма, как, например, в недавней работе по моделированию головастика Xenopus. Однако наибольший интерес, как практический, так и фундаментальный, представляют организмы, обладающие зрением, более сложной нервной системой и, соответственно, значительно более развитыми когнитивными способностями. Определить структуру и параметры нервных систем таких организмов представляется исключительно сложной задачей. Бо- лее того, они изменяются с течением времени, в том числе под воздействием воспринимаемых ими потоков сенсорных сигналов и полученного жизненного опыта, включая последствия собственных действий при тех или иных обстоятельствах. Зная структуру нервной системы и число образующих ее нервных клеток хотя бы прибли- зительно, можно попытаться оптимизировать начальные параметры модели посредством искусственной эво- люции, в процессе которой виртуальные организмы будут взаимодействовать и выживать – каждый под управ- лением собственной версии нервной системы. Помимо этого, эволюционировать могут и правила, по которым мозг изменяется на протяжении жизни организма. Данная работа посвящена созданию нейроэволюционного симулятора, способного осуществлять одновременное функционирование виртуальных организмов, обладаю- щих зрительной системой, которые взаимодействуют между собой. Приведены расчеты, показывающие, сколь- ко вычислительных ресурсов требуется для работы моделей физического тела организма, нервной системы и виртуальной среды обитания, а также определена производительность симулятора на современной настольной вычислительной системе в зависимости от числа одновременно моделируемых организмов.

## Introduction

Computational models imitating the functioning of living organisms’ nervous systems, based on their electrophysiological and morphological data, are powerful tools in neuroscience. With their help it is possible, on the basis of knowledge and ideas about the functioning of individual nerve cells and the mechanisms of interaction between them, to calculate the dynamics of the activity of networks of nerve cells. The model of the nervous system functioning in combination with the model of the body of an organism equipped with muscular and sensory systems, placed in a virtual three-dimensional physical environment, provides the researcher with significant advantages. First, one can observe and register both the behavior of the body model of an organism and the activity of the nervous system, up to the activity of individual nerve cells, their processes and synapses. Secondly, the model of the nervous system receives a stream of signals from the virtual environment that change in response to the actions of the organism, driven by a muscular system controlled by its “brain”, i. e. there is a constant feedback between actions and their consequences, just like in reality. One of the goals of such modeling is to check the adequacy of neural network models by comparing the activity of nervous systems of a real organism and its virtual ‘twin’, as well as their behavior.

Probably the most well-known creature in this context is one of the most simple multicellular organisms, invertebrate Caenorhabditis elegans, whose nervous system is composed of just 302 neurons (Sarma et al., 2018). Also, sufficiently convincing similarity between the real organism and the model was achieved for the Xenopus frog tadpole at the two-day stage of development, whose nervous system model was represented by a neural network composed of approximately 2300 neurons (Ferrario et al., 2021). However, neither C. elegans, nor the two days old Xenopus tadpole has a visual system.

Attempts to model much more complex organisms such as a mouse (~70 million neurons (Herculano-Houzel et al., 2006)) or a rat (~200 million neurons (Herculano-Houzel, Lent, 2005)), including their nervous systems, have also been made. However, to date, their virtual twins have not yet been created. The work aimed at reverse engineering and modeling the nervous system of the Drosophila fruit fly (~100 thousand neurons (Scheffer et al., 2020)) is also in progress. Another extremely promising object of investigation and modeling is ants (~250 thousand neurons (Moffet et al., 2021)). These insects have immobile compound eyes, consisting of 100…3000 ommatidia – structural and functional units of such eyes (their number depends on the type of ant and its specialization), providing color vision with a rather modest resolution (from 10×10 to 55×55 “pixels”). Thus, for example, the eyes of Myrmica ruginodis usually have 109 to 169 ommatidia, and those of Camponotus crassus and Pseudomyrmex adustus, which are active during daylight hours – up to 700 and 930, correspondingly (Aksoy, Camlitepe, 2018), and the maximal known number of ant ommatidia per eye, near 3000, was registered in tropical species Gigantiops destructor (Macquart et al., 2006).

It is noteworthy that ants are the simplest organisms that successfully pass the mirror test, i. e. they are able to distinguish their own reflection in a mirror from another ant, which they can see through ordinary transparent, non-mirror glass of the same size (Cammaerts M.-C., Cammaerts R., 2015). The principle of conducting a mirror test is worth mentioning. In front of a mirror, ants clean themselves up or make unusual movements of their head and antennae, which is not observed when they see relatives behind the glass. If a small mark (e. g. blue) is applied on the front of an ant’s head, then when it sees itself in the mirror, it will try to get rid of it, try to clean it off with the help of its legs. And if the mark is the same color as the body of the ant, or if it is applied to the back of the head, not visible in the mirror, then the ant will not show concern and attempts to clean it off. Thus, the ants notice the mark on themselves and behave as if they understand that it is on themselves, and not on another ant, relying solely on visual signals.

Computational modeling of both a single ant, with or without a mark, able to see itself in a mirror, as well as multiple ants that can see and interact with each other and with surrounding objects is of considerable scientific interest. Orientation on the terrain in ants is also carried out mainly through vision (Buehlmann et al., 2020).

What are the requirements for a software system and computing hardware capable of performing computer simulation of a group of virtual organisms imitating ants (including body, muscle, sensory and nervous systems) and their habitat? It is assumed that organisms can interact with each other in the physical world and “see” each other, i. e. their nervous system receives a stream of video data corresponding to the first-person view as input. The problem of “digitizing” the structure of the nervous system, including 3D morphology of each neuron, its processes and synapses, is extremely laborand time-consuming. However, this may not be essential, since the brain, even in ants, is quite plastic and undergoes noticeable structural changes during the life of the organism (Penick et al., 2021). At the same time, not much is known about the mechanisms underlying brain changes throughout life at the level of single neurons and connections between them. Therefore, it makes sense to pose the problem of modeling an organism that has the body and sensory systems of an ant (at least visual and mechanosensory, as well as the simplest olfactory and taste receptors) and a nervous system with a similar number of neurons and synapses, but without a fixed connectome. How fast can such modeling be carried out and can one expect that virtual evolution in such a system will help artificial neural networks to achieve cognitive capabilities that will allow virtual organisms to effectively survive, solving more or less complex tasks related to finding food, avoiding hazards and performing other activities?

## Materials and methods

Software system. In accordance with the subject of the article, we are using computational modeling to deal with the problems to be solved – the research is carried out based on the software that we developed for conducting numerical experiments in the field of neuroevolutionary modeling. It is based on a modern 3D physics engine named Unigine (unigine.com), which is used for developing games, virtual reality systems, interactive visualization software, educational systems in various areas, etc., supporting Windows and Linux platforms.

The physics simulation module supports collision detection, rigid body physics, various types of joints (hinged, ball, prismatic, cylindrical, etc.), dynamic destruction of objects, cloth, floating objects, force fields, time reversal, etc. (https:// developer.unigine.com/ru/docs/latest/principles/physics/). In Unigine it is possible to use mirrors, which may be useful in the future for conducting a “mirror test”. Also, it has built-in C++ programming language, which allows to develop and use one’s own program code – for example, to model networks of neurons that receive signals from virtual organisms sensory systems and control their movements

**Fig. 1.**
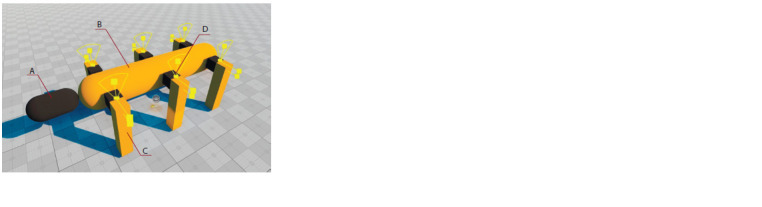
Simple “ant” 3D body model, general view. А – head, B – body, С – legs, D – a joint connecting body and legs. The head has a movable connection with the body.

An “ant” body model. The simple “ant” body model that we designed and used as a first prototype to evaluate the performance of the simulator is shown in Figure 1. In the future, it is planned to develop and use a much more detailed and realistic version

**Fig. 2.**
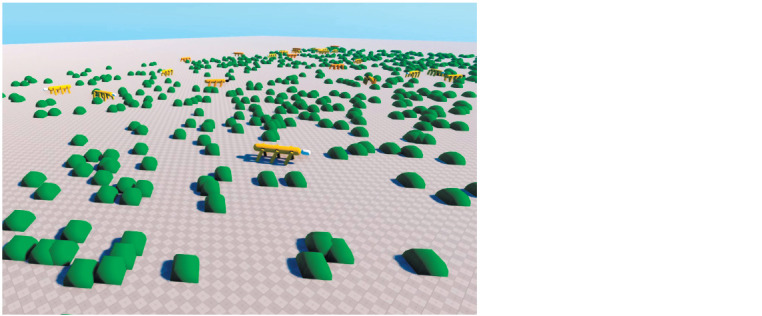
General view of the simulation – test scene with a few dozens of virtual organisms.

In the simplest test scene, food particles (shown in green) and several dozen virtual organisms are randomly placed on the plane (Fig. 2).

**Fig. 3.**
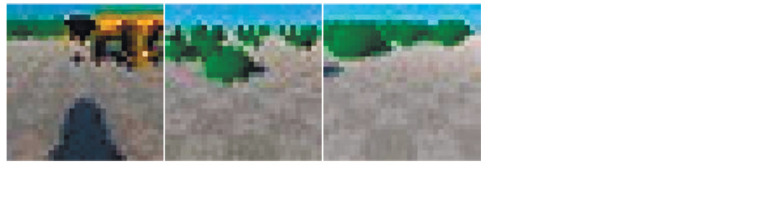
A few examples of the “first-person view”. In the first one (on the left), one can distinguish another individual (top, in brown tones) and the shadow of the virtual organism perceiving this image (dark gray).

Visual system. Figure 3 shows examples of images perceived by a “video camera” located on the body’s head, which is directed forward (at the moment only color mono-vision is implemented, although stereo is also planned for the future). The resolution of frames of ant’s video stream was chosen to be 30×30, which approximately corresponds to the average spatial resolution of visual systems of real ants considered earlier. Since the images themselves are quite small, for the convenience of perception in the figure they are proportionally enlarged by 5 times (one color square of 5×5 pixels corresponds to one real “receptor” pixel).

An image can be represented as three matrices, each of which represents a separate color channel (red – R, green – G and blue – B). Each matrix has a size of 30×30, forming an array of data, Input, consisting of 2700 elements, organized in the following way:

**Formula. 1.**
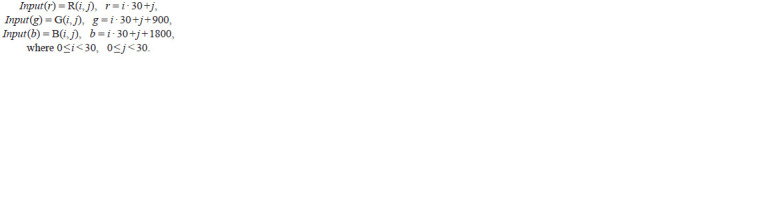
Formula. 1.

The simulation has a certain frame refresh rate, depending on the computational performance of the hardware, the complexity of the simulated scene and the number of “ants”. With a certain frequency, each individual forms such an array, the content of which enters the “nervous system” of the organism.

Nervous system. Visual signals enter “nervous systems” of virtual organisms, which at the very beginning of the simulation, for the first generation of “ants”, are randomly generated networks of artificial neurons, similar to those used in perceptrons (Rosenblatt, 1962) for recognition of letters, digits and geometrical figures. In our case, the number of neurons in each network was about 3000. Within the lifetime of one individual, networks have a static topology. Perceptron consists of S-elements (sensory), one or more layers of А-elements (associative) and R-elements (reacting). А-elements are defined by a set of weight matrices А1, А2, …, Аn and bias vectors b1, b2, …, bn. The array Input, mentioned above, is processed in the following way:

**Formula. 2.**
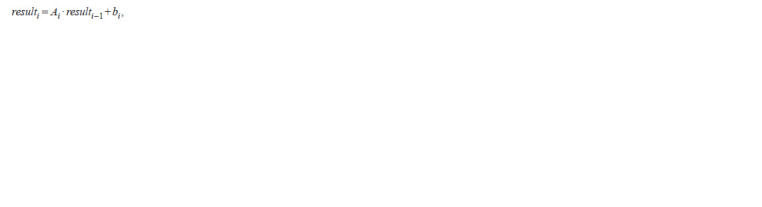
Formula. 2.

where result0 is a layer of sensory elements, containing an array of visual data perceived by an “ant”, and i = 1,…, n. And activation of R-elements as a result of visual data processing leads to the corresponding actions performed by the ant (change of speed, turn to the left or to the right).

Simulation of evolution. Some variants of weights matrices of perceptrons described above provide more efficient survival, i. e. the ability to perceive “first-person view” visual signals, analyze them and control the movement of the body in such a way that an organism regularly reaches food particles and maintains the necessary “energy level” in the body (satiated state). Organisms that remain hungry for too long die out and the “long-livers” have the opportunity to generate offsprings that inherit the structure of their neural networks. Currently, offspring is generated by only one parent (in nature such a reproduction mechanism, called parthenogenesis, also exists – in many types of arthropods, including 8 species of ants, as well as in about 70 species of vertebrates).

In the simulator, the current “energy level” of the organism is indicated as Satiety (t), with which the following quantities are associated:

MaxSatiety – maximum organism satiety (15 by default); BirthSatiety = MaxSatiety · 0.7 – the satiety of the organism, upon reaching which it gives birth to a descendant. When it happens, half of the available resources remains with the organism, and half passes to the descendant.

Each organism is initialized with Satiety (0) = 8. Each time after a certain period, it loses one satiety unit (because organism functioning “consumes energy”). At Satiety (t) = 0 the organism dies. When eating food, the organism gains a satiety point until MaxSatiety is reached.

The child inherits the parent’s neural network with changes that are carried out according to the following rules: ε, δ – random values which are distributed uniformly; ε [a, b] – probability of changes in neuron parameters (“mutation”), 0 ≤ a ≤ b ≤ 1; δ [c, d ] – the amount of weight change in the matrix element as a result of mutation, c ≤ d. Parameters a, b, c and d can be changed by user. Every element of weight matrices and bias vectors, Ak (i, j ) and bk (l ) (k = 1, …, n) changes by +δ or –δ with probability ε.

## Results

At the current stage of the work, the main achieved result is the development of the simulator prototype (including a three-dimensional physical world, a model of the physical body of an ant, a model of the visual system and a model of the nervous system), as well as measurements of its performance on various computing systems, depending on their characteristics and on the number of neurons in the nervous system of virtual organisms. The source code of the simulator is available in the following repository (https://github.com/ NotNa19/AntPrototype). Perspectives of further development of this project depend on the ability to perform neuroevolutionary modeling for at least one, but preferably for more virtual organisms, whose “nervous systems” are comparable to those of real ants in terms of the number of nerve cells.

Table 1 contains the characteristics of the computational hardware used in the testing and the maximum number of virtual organisms modelled simultaneously for which the simulation still remains stable. In this case, “stable work” means the correct functioning of organisms and their physical bodies. The fact is that in the current version of Unigine, at a low frame rate, delays between the movement of various components of the organism may occur, the processing of collisions between the objects, including “organisms” and “food”, may not always work correctly, and some other problems of this kind may happen as well. It is possible to fix these problems and it is planned for the future, but it requires a deeper knowledge about the mechanisms of the 3D engine. With a screen resolution of 1920×1080 pixels and its refresh rate (frames per second, FPS) of at least 30 per second, the simulator remains stable. However, the number of individuals simulated at the same time affects the performance. The following values were obtained on our computational hardware:

**Table 1.**
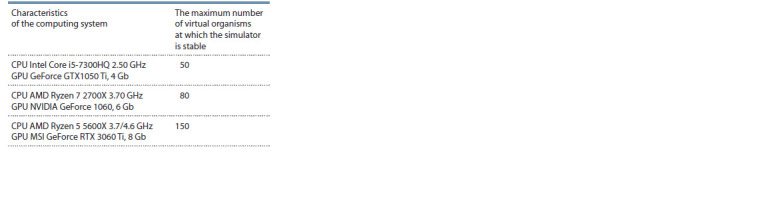
The maximum size of the population of virtual organisms at which the simulator is stable, depending on the characteristics of the hardware used

Detailing of the time spent on various stages of the simulation showed that with a small size of nervous systems (thousands to tens of thousands of neurons), the most significant factor limiting the speed of its operation is the process of obtaining “first-person view” video stream data for the ant population, even considering the fact that the multithreading of calculations is provided by the engine itself. Dependence of the maximum number of individuals in the simulation on the number of neurons in the “nervous system” of the virtual organism (all individuals have the same number) has also been investigated. The following values were obtained for GeForce RTX 3060 Ti + AMD Ryzen 5 5600X (Table 2).

**Table 2.**
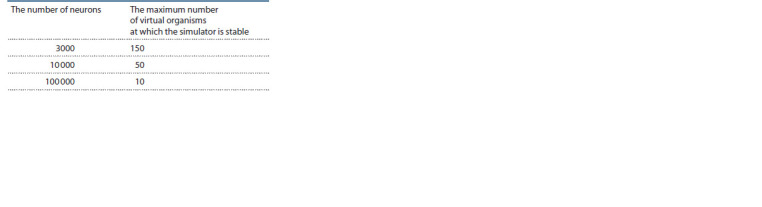
The maximum population size of virtual organisms at which the simulator is stable, depending on the number of neurons in their “nervous system”

The costs of 3D scene visualization for an external observer also have a noticeable impact on the performance of the system. Measurements performed at the computational system composed of AMD Ryzen 7 2700X 3.70 GHz CPU and NVIDIA GeForce 1060 6 Gb GPU revealed the following:

• When performing a simulation with an empty scene (with or without visualization for an external observer), stable 9000 clock cycles in 60 seconds (an average of 150 clock cycles/sec) are obtained. • When performing a simulation with 80 organisms, with visualization for an external observer, we get 5400 cycles in 60 seconds (an average of 90 cycles/sec), and 7800 cycles in 60 seconds (an average of 130 cycles/sec) without visualization. • With a higher load (100 individuals and more food), we obtained 1800 cycles in 60 seconds with visualization (on average 30 cycles/sec) and 4500 clock cycles in 60 seconds without visualization (an average of 75 clock cycles/sec).

Thus, visualization for an external observer (user) plays a fairly significant role in the overall performance of the system and thus it makes sense to turn it on only when it is really necessary – for example, in cases of debugging or recording demo videos illustrating the functioning of the simulator.

**Fig. 4.**
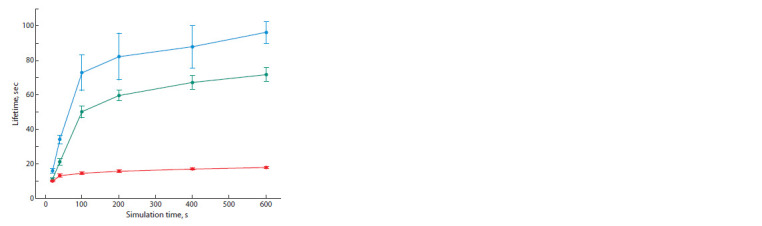
The dependence of the maximum lifetime of an individual from the population for the entire period from the beginning to the present moment (blue curve), at the moment (green curve), and during the average lifetime of the population (red curve), indicating the root-meansquare deviation. The data is obtained from 10 simulation runs.

The work of the genetic algorithm can be illustrated by the dependence of the individuals’ lifetime, which increases as the number of generations grows. The curves shown in Figure 4 were obtained based on 10 runs of the simulator with the same parameters

It can be seen that over time there are individuals appearing in the population whose lifetime is many times longer than the lifetime of individuals with randomly generated neural network parameters that have not yet passed natural selection. At the behavior level and with visual observation, it is expressed in the fact that the most adapted virtual organisms purposefully move towards the particles of “food” and avoid moving away from the central area of space with the largest concentration of “food”, i. e. they are successfully adapted to their living conditions.

## Discussion

The current neural network architecture is quite simple and at this stage was used mainly for testing the system as a whole and for evaluating its performance at an early stage of development. Currently, the following much more advanced and modern neural network architecture, which is a combination of a convolutional neural network (LeCun, Bengio, 1995) (for working with incoming video data) and the NEAT algorithm (NEuroevolution of Augmenting Topologies) (Stanley, Miikkulainen, 2002) is being implemented. NEAT can change not only the weight parameters, but also the structure of the neural network during the lifetime of the organism. The convolutional neural network will transform the details of the image to some abstractions, and the NEAT algorithm will handle the behavioral part of the virtual organism and work with the results of the functioning of this convolutional neural network

In addition to this variant, self-organizing networks such as neocognitron (Kunihiko, 1980) are quite promising in terms of architecture as well. There are also neural networks that are much more realistic in terms of electrophysiology and neuromorphology. They are based on the Hodgkin–Huxley nervous cell model (Hodgkin, Huxley, 1952), in which it is represented in the form of compartments characterized by electrical capacitances and resistances, with calculations of membrane potentials and ion currents. The modern implementation of this model with support of parallel computing on GPUs has the following performance indicators. In the work (Stimberg et al., 2020), a neural network of 64 thousand neurons required about 0.6 sec of working time on a Tesla V100 GPU (with a performance of 14.1 TFLOPS in FP32 mode) to calculate 1 sec of simulation time (i. e. real time), and about 3 sec of calculations per 1 sec of simulation time – for neural networks of 256 thousand neurons. At the same time, numerical integration of the equations describing the system occurs with a time interval not exceeding 0.1 msec to ensure the accuracy of calculations and stability of the system, and each neuron on average has about 1000 connections (80 % of which are activating, and 20 % are inhibiting).

Recently, the research on new neural network architectures has been quite actively conducted, and many of obtained results have been successfully applied in practice. Particularly, in the field of neuroevolutionary methods, quite a wide range of promising variants has been considered, classified and compared in the dissertation (Khlopkova, 2016, Ch. 1) and in the review article (Ma, Xie, 2022). In the future we plan to implement the most suitable and promising of them in the presented simulator and explore the limits of their “cognitive capabilities” while controlling the virtual “ants”.

## Conclusion

Modern GPUs, such as, for example, NVidia 3080 Ti, with 10240 parallel CUDA computing cores, have a performance of 34.1 TFLOPS, and the upcoming 4080 Ti is expected to have 67.6 TFLOPS. Thus, the technological capability to simulate a single virtual organism with a biologically realistic neural network of 256 thousand neurons and 256 million connections between them, with a numerical integration time step equal to 0.1 msec, on a single GPU, has already been achieved. It is comparable to the neural network of the real ant’s nervous system, which includes about 250 thousand neurons.

Our calculations for virtual organisms with neural networks of several thousand elements have shown that the computational costs of neural networks and the virtual physical environment are relatively small, and the main limiting factor for the system performance is video data streams in the “first person view” mode, carrying visual information. However, in the case of neural networks consisting of hundreds of thousands of neurons, the “nervous system” becomes the main consumer of computing resources. Thus, given the above, a modern desktop computing system with a powerful modern GPU has enough performance to provide a real time simulation of a virtual organism with a “nervous system” based on the Hodgkin–Huxley model, with a number of neurons composing its nervous system equivalent to that of a real ant. And if there are multiple GPUs in one workstation, the number of simultaneously simulated ants interacting with each other can be increased in proportion to the number of GPUs.

## Conflict of interest

The authors declare no conflict of interest.
